# Survey data on factors that constrain the adoption of soil carbon enhancing technologies in Ethiopia

**DOI:** 10.1038/s41597-020-0431-9

**Published:** 2020-03-17

**Authors:** Wilson M. Nguru, Stanley K. Ng’ang’a, Fekadu Gelaw, George M. Kanyenji, Evan H. Girvetz

**Affiliations:** 1 Department of Land Resource Management & Agricultural Technology, University of Nairobi, P.O. Box 29053-00625, Kangemi, Kenya; 2 International Center for Tropical Agriculture (CIAT), P.O. Box 823-00621, Nairobi, Kenya; 3 Haramaya University, Ethiopia, P.O. Box 138, Dire Dawa, Ethiopia; 4 Department of Agricultural Economics, University of Nairobi, P.O. Box 29053-00625, Kangemi, Kenya

**Keywords:** Socioeconomic scenarios, Agriculture, Climate-change mitigation

## Abstract

The data described in this paper were collected in two watersheds – Azuga-suba watershed in Angacha woreda (district) of Southern Nations, Nationalities and Peoples (SNNP) region and Yesir watershed of Bure woreda (district) of the Amhara region – in Ethiopia. The data were collected from 379 households with the main objective being to assess the factors constraining the adoption of sustainable land management technologies (SLMT) that enhance soil organic carbon sequestration. The data were collected using a structured questionnaire that was designed in SurveyCTO. The data cleaning and analysis was done using STATA SE version 14. This data can be used by researchers to assess the extent of adoption of SLMTs in Ethiopia by, for example, comparing the North and the South regions of Ethiopia. It can also be used to assess the probability of adoption as well as the benefit and costs of adopting SLMTs in Ethiopia both at farm and plot level.

## Abstract (summary)

**Table:** 

Measurement(s)	Socioeconomic Factors • Access • wealth • plot specific information • agricultural process • forestry • soil conservation activity • Demographics
Technology Type(s)	Survey
Sample Characteristic - Environment	farm • farm soil
Sample Characteristic - Location	Ethiopia

Machine-accessible metadata file describing the reported data: 10.6084/m9.figshare.11830887

## Background & Summary

Soil degradation is the main reason for the continued poor agricultural productivity in sub-Saharan Africa (SSA)^1^ with degraded agricultural land accounting for 65%. This is mainly due to low nutrient application, soil erosion, acidification and sodification^2^. With the population of the SSA projected to double by the year 2050^3^, soil degradation and low agricultural productivity pose a threat to the human population. Soil degradation is attributed to reduced soil organic carbon (SOC)^4^ that enhances soil fertility by improving soil water holding capacity, cation exchange capacity & soil aggregation and reducing the soil susceptibility to crusting and soil erosion^5,6^. Loss of SOC is mainly attributed to land-use changes especially the conversion of natural vegetated lands such as forests and grasslands to cultivated land. Evidence from published research shows that conversion of marginal lands may not lower SOC stocks, instead, application of sustainable land management technologies (SLMTs) that encourage constant retention of stubble in the soil may lead to the build-up of SOC at a high percentage with time^7,8^.

Sustainable land management technologies (SLMTs) ensures sustainable agricultural production and livelihoods, in the long run, improving the standards of living of households and improving agricultural productivity^9–11^. Developing countries have been making constant efforts to increase farmers’ adoption of SLMTs. In Ethiopia for example, programs such as Poverty Reduction Strategic Paper I, Plan for Accelerated and Sustained Development to End Poverty (PASDEP), Agricultural Transformation Programs (ATP), Agricultural Growth Program (AGP), Production Safety Net programs (PSNPs), Food Security programs, and many other programs and projects have been implemented over the past decades^12–15^. Despite these efforts, adoption of SLMTs has remained low and farmers continue to practice conventional farming that leads to the continued depletion of SOC with continued soil degradation affecting agronomic productivity^5^.

Past research on the factors affecting the adoption of SLMTs in Kenya and Ethiopia show that factors that constrain adoption of SLMTs include farming technologies adopted by households, agro-ecological variations, plot characteristics, farmer social-economic characteristics and institutional assistance^16–18^. Social-economic characteristics that are known to affect the adoption of SLMTs include; education level of the household head, farming experience, gender, plot size, tenure security, membership in farmer groups, agricultural extension, distance to plots, market access, credit access, livestock ownership and household size^19,20^ while biophysical characteristics include, plot slope, rainfall, soil type and soil erosion^21^. Through the years of constant effort-making from the government, non-governmental institutions and individual efforts, farmers have adopted the SLMTs at a lower percentage. This study seeks to find out the extent that farmers have adopted the SLMTs and the factors that constrain the adoption. The data described in this paper were used to evaluate the extent of adoption of SLMTs and what constraints their adoption. The data contains information on socio-economic factors, plot-specific characteristics, institutional factors, wealth ranking, marketing, and information relating to the access to infrastructure that supports farmers’ development^22^. The data also contains information on the types of crops grown and animals kept, their productivity and marketing.

The survey tool that was used to collect the data was reviewed thoroughly and approved by the Internal Review Board (IRB) of the International Centre for Tropical Agriculture (CIAT) before the study was carried out similar to^22^. In addition, each farmer was asked to sign a consent form as an indication that they were willing to be interviewed. The sampled areas represented the highland areas of Ethiopia characterized by an annual rainfall of above 1000 mm and where farmers practice mixed cropping and livestock farming systems. Therefore, this data can be used in a broader category to assess the dimensions of household financial, human, physical and social capital in the study sites and other areas with similar characteristics to the study sites as represented. It could also be used for comparison with similar household-level surveys in other study sites. In a similar study as was used in this research, the data can be utilized to carry out further research on the extent of adoption of specific SLMTs, the probability of adoption in the study, reasons behind farmers’ decision to implement particular SLMTs, and benefits and costs of adopting SLMTs by farmers.

## Methods

### Data collection

**Fig. 1:**
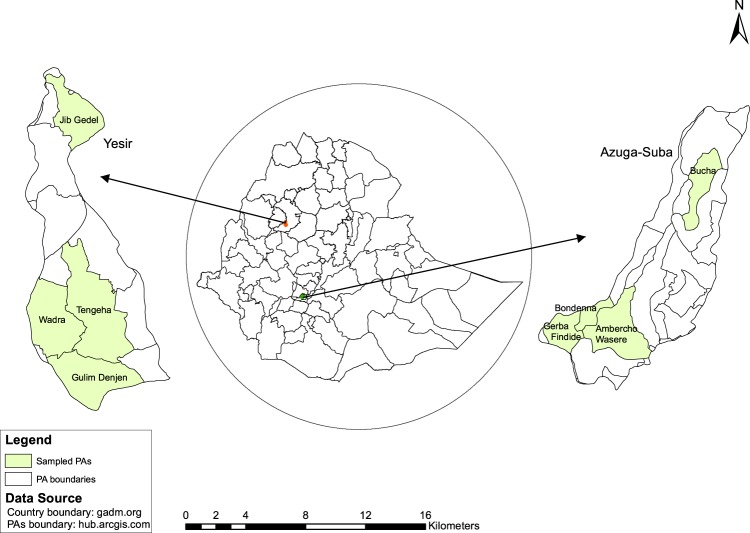
A map of Ethiopia showing the studied watersheds and the sampled pastoral areas.

The data were collected from households drawn from two watersheds Yesir and Azuga-Suba. Yesir watershed is located in Bure district (Woreda), Western Gojjam Administrative Zone in Amahara Region, while the Azuga-Suba watershed is located in Kembata Tembaro Woreda, and Wolaita Administrative Zone in the Southern Region (Fig. 1).

A two-stage, multistage sampling technique was utilized to generate the sample. The central aim of the sampling design was to obtain households that were representative of the various household groups and landscapes of the two watersheds. In the first stage, the Kebele Administrations (*also referred to as* Pastoral associations (PA) which is the lowest administrative structure in the watershed (a watershed comprised of several PAs) were categorized into three zones: PAs in the upper zone, middle and lower zones. In line with this, one PA on the upper zone, two PAs in the middle zone and one PA from the lower zone of the watershed were randomly selected from the existing PAs in each category summing up to four PAs per watershed. Two PAs were selected in the middle zones due to high population distribution in these zones. Accordingly, households that have a plot of land within the watershed in the four selected PAs were used as the sampling frame. In the second stage, and using this sample frame, the numbers of sample households in each PA were allocated as a proportion to the number of households in the PAs. Accordingly, 161 households in the four PAs in Azuga-suba watershed and 218 households in the four PAs in Yesir watershed were drawn randomly from the PAs household roster which gives a total sample of 379 households. The selected households represented various levels of wealth categories with different socioeconomic and biophysical characteristics. In order to avoid missing responses, only those households that had a plot of land in the watershed were included in the sampling. When the selected household was not accessible, he/she was replaced by the next household in the list. The distribution of sampled households across watersheds and PAs was as summarized in Table 1.

**Table 1:** Distribution of sample households in the two watersheds.

Woreda/district	PA	N	%
Azuga-Suba	Ambercho Wasere	47	12.4
	Bondenna	46	12.1
	Bucha	23	6.1
	Gerba Findide	45	11.9
	***Sub-total***	***161***	***42.5***
Yesir	Gulim	51	13.5
	Jib Gedel	69	18.2
	Tengeha	44	11.6
	Wadra	54	14.2
	***Sub-total***	***218***	***57.5***
Grand total		379	100

Household data from the sample households were collected using a structured questionnaire. The main focus of the questionnaire was key household characteristics that are known and/or expected to influence the adoption of SOC enhancing land management practices and included detailed questions on the various SOC enhancing practices. The enumerators involved in this study were trained for one day followed by pre-testing of the tool to ensure that they understood the questions properly as well as how to key in SurveyCTO. The enumerators were also made aware of the common mistakes that may occur during the survey. Data collection was carried out by six enumerators in each watershed. The collected data was checked by the supervisors on a daily basis and appropriate correction measures were taken.

## Data Records

This data are available as.dta, Rdata or as tab-delimited. .dta opens in Stata and from it can be converted into other formats such as .csv or.xls formats, Rdata opens in R while tab-delimited format can be opened in excel and converted into .csv or xls formats. Missing values were identified with a dot, which is the universal manner of denoting missing values in Stata. The data is stored in the Harvard Dataverse Repository under the International Center for Tropical Agriculture (CIAT) Dataverse (CGIAR)^23^.

The data were arranged as in the survey questionnaire that was divided into six sections containing 10 parts namely; household characteristics, details about household demography, household wealth indicators liquidation, household’s access to infrastructural services, plots of land owned and operated, input use, soil conservation and agroforestry activities, access to output markets, access to credit and access to extension services. The sections were Household characteristics, Plot level data, About carbon enhancing practices, Market participation and credit access, Social capital and contact information. The introductory part involved the collection of general information of the study area. Part one of section one covered household characteristics including the respondent’s name, gender, place of birth and years of farming for the household head. Part two gave the details of all household members; age, schooling, work, marital status and participation in farming activities. Part three collected details on wealth indicators including livestock holding while part four collated information about household’s access to infrastructures.

Section two contained two parts: parts five and six. Part five contained plot-level data such as the number of plots owned, the number of plots operated in 2017, size of each plot, type of crops grown on each plot, farm activities, yield per crop, residue management, soil type, slope, distance of the plots from the households, soil erosion, soil and water conservation activities and land ownership. Data on input use, quantity, and purchasing price were contained in part 6.

Section three contained only part seven with information on soil and water conservation, agroforestry activities and factors enhancing soil organic carbon.

Section four contained part eight to 10. Part 8 had details on farmers’ access to output markets, crops sold, their quantities and their market prices, while part nine contained data on access to credit services. Part 10 contained details on access to extension services. This was followed by section five and six.

In this survey, the household head was targeted but in case of his/her absence, other respondents such as the wife or any other respondent with over 18 years of age and more than five years’ experience in farming were interviewed. Table 2 below shows the description of the dataset as per the questionnaire with the themes that were generated from the questionnaire^22^.

**Table 2:** Description of the dataset as per the questionnaire. The questionnaire generated six themes: Socio-economic factors (SEC), Access to infrastructure (INF), Wealth information (WEI), Plot specific information (PLI), Agricultural practices and activities (AGRA), and Access to external support services (ESS).

Variable	Theme	Description	Duration Considered
Demographic	SEC	Gender, age, education level, marital status and occupation of the household members	12 months
Infrastructure	INF	Distance in walking minutes to an all-weather motorable road, winter motorable road, asphalt road, nearest rural market, nearest town market, electricity, health post, and health center.	Current
Wealth Index	WEI	Likelihood of a family unit not being poor as guided by 10 questions.	Current
Plot specific Information	PLI	Indicates the plot size, tenure system, if its irrigated, type of crop, previous crop, residue use, manager of the plot, the distance of the plot from the homestead, their slope, soil type and farmers perception towards their soil fertility, use of inputs and soil erosion	Current
SLMP	AGRA	Soil management practice implemented in each plot, period, the purpose of adoption and challenges associated with each practice	Current
Crop Yield	AGRA	Crops that are grown by the farmers and their yield in the last 2 year	The last 2 season
Crop Market Participation	AGRA	Crop produce sold by farmers, the quantity and the revenue received	Last 12 months*
Input	AGRA	Indicates if farmers used inorganic or organic fertilizers, the amount used, source and price per unit if purchased	Last Season
Labour	AGRA	Labour sources for the farming activities	The last 2 season
Livestock	AGRA	Indicates the number of livestock owned	Last 12 months
Livestock Market Participation	AGRA	Indicates how many animals were sold in the past 12 months and the price per animals.	Last 12 months
Social Capital	ESS	If the farmers belonged to any farmer group or organization the type of the group, membership fee and their main role in the group.	Last 12 months
Access to Credit	ESS	If any household member acquired a loan, type of the loan, amount received and purpose of the credit	Last 12 months
Access to Extension	ESS	If farmers accessed extension advice or training, source, kind of extension, application on-farm and the usefulness of the information acquired	Last 12 months
Sources of Income	WEI	The different source of income that the household has	Last 12 months

*The survey was conducted in September 2018 which is the first month of the Ethiopian Calendar (Meskerem) and the end of the second season of the year.

## Technical Validation

The data described above are cross-sectional and were obtained by interviewing individual farmers. For plot level biophysical and social-economic information, it was difficult to ascertain the ability of the tool to collect quality information as most of it was based on farmers’ perceptions and proxies. For example, in plot-level information as presented in Table 2, plot sizes were obtained from the farmers’ perception of the sizes of each plot and in some cases the enumerators had been trained to ask the total farm size and divide it into the number of plots in the farm, in case the farmer did not know the size of their plots, this would be provided in local units which would later be converted into standard units. Tenure security was represented by the farmer owning a title deed, distance to the plot would be a farmer estimation of the number of minutes they would take to walk to a specific plot, the plot slope was divided into two categories, plain and sloppy while the soil type was divided into three categories, loamy, sandy and clay and enumerators trained to differentiate each by looking at it or by the farmers colour description.

It is normal for this sort of information to have a few issues, for example, missing data or under or over-reporting. To correct this challenge, SurveyCTO was utilized whereby all the required information was captured exhaustively by giving options of choice as well as multiple categories covering all variables anticipated to be collected. This involved conducting focus group discussions and key informant interviews before the tool development and coding in the SurveyCTO, to ensure that responses to each question were captured well. The over and under-reporting of various variables such as output and price were also checked against the average outputs in the region as well as the prevailing market prices. Addition information was also collected on notebooks and later incorporated into the tool to enhance the quality of data.

## Usage Notes

The data is available in Stata data format and can be opened using Stata program Version 13 and above. In case a user has an older version of Stata, a means of converting the data to a compatible form is provided in Stata. For those who do not have access to Stata software, Statistical Package for Social Sciences (SPSS) software can also be utilized to open the data, under the import tab provided they specify that the data is in Stata data format (.dta). In addition to this, R an open-source software can also be used by exporting this data to comma-separated values (.csv) and importing it into R. The questionnaire that was used to collect the data is also provided together with the data to facilitate ease of understanding of the data.
